# Alveolar-derived autologous blood clot as a scaffold for regenerative endodontic treatment: An observational study

**DOI:** 10.6026/973206300212486

**Published:** 2025-08-31

**Authors:** Gopikrishnan P.B., Rajeswari Putchala, Vankala Gautam, Aparna Srivastava, Rahul Tiwari, Heena Dixit, Deepak Sharma

**Affiliations:** 1 Department of Conservative and Endodontics, AL -AZHAR DENTAL COLLEGE, Thodupuzha, Kerala, India; 2 Department of Conservative & Endodontics, ST. Joseph's Dental College, Duggirala, NTR University of health sciences, Andhra Pradesh, India; 3 Department of Conservative Dentistry and Endodontics, Buddha Institute of Dental Sciences and Hospital, Patna, Bihar, India; 4 Department of Oral and Maxillofacial Surgery, RKDF Dental College and Research Centre, Sarvepalli Radhakrishnan University, Bhopal, Madhya Pradesh, India, India; 5 Department of Hospital Administration, Index Institute of Management, Arts and Science, Malwanchal University, Index City, Nemawar Road, Indore, Madhya Pradesh, India

**Keywords:** Regenerative endodontics, blood clot scaffold, immature teeth, apical closure, root development

## Abstract

Regenerative endodontic treatment (RET) has transformed the management of immature necrotic permanent teeth by promoting biological healing and root development. Hence, a total of 20 patients aged 8-16 years with necrotic immature anterior teeth were enrolled. Blood was collected from a fresh alveolar socket and introduced into the canal space following canal disinfection with triple antibiotic paste. 83.3% showed complete periapical healing, while 72.2% exhibited apical closure and root lengthening by 12 months. We further found that clinical signs such as pain, swelling and sinus tract were resolved in all cases.

## Background:

Regenerative endodontic treatment (RET) has emerged as a viable biological alternative to conventional apexification techniques in managing immature permanent teeth with necrotic pulps. Traditionally, calcium hydroxide and MTA have been used to induce apical closure; however, they do not support continued root development or restore the natural pulp-dentin complex [1, 2]. In contrast, RET aims not only to eliminate infection but also to re-establish vitality by stimulating the body's own healing capacity through tissue engineering principles, namely the triad of stem cells, scaffolds, and growth factors [3]. Among these, the scaffold plays a critical role in providing a three-dimensional matrix for cell migration, attachment and proliferation. Various materials have been explored as potential scaffolds in RET, including "platelet-rich plasma (PRP)", "platelet-rich fibrin (PRF)", and synthetic biomaterials [4]. However, their cost, technique sensitivity and variability in handling have limited widespread clinical adoption. In this context, the use of an autologous blood clot specifically derived from the alveolar bone cavity during treatment as a natural scaffold offers a cost-effective, biocompatible and minimally invasive alternative [5, 6]. The alveolar-derived blood clot is rich in endogenous growth factors, including "vascular endothelial growth factor (VEGF)", "platelet-derived growth factor (PDGF)" and "transforming growth factor-beta (TGF-β)", which may facilitate angiogenesis and stem cell homing within the root canal environment [7]. Furthermore, as an autologous product, it poses minimal risk of immunogenic reactions or disease transmission. Despite its theoretical advantages, clinical data evaluating the efficacy of this approach remain sparse. Emphasis is placed on periapical healing, continued root development and apical closure over a follow-up period, aiming to validate its potential as a simple and biological scaffold in clinical RET protocols [8, 9-10]. Therefore, it is of interest to investigate regenerative outcomes using alveolar-derived autologous blood clot as a scaffold in necrotic immature permanent teeth.

## Material and Methods:

## Research design and ethical approval:

This was a single-center observational research conducted in the Department of Conservative Dentistry and Endodontics over a period of 18 months. Ethical clearance was obtained from the Institutional Ethical Committee (Approval No: IEC/END/2023/08), and written informed consent was taken from all participants or their guardians in the case of minors.

## Sample selection:

A total of 20 patients aged between 8 and 16 years with necrotic immature permanent maxillary or mandibular anterior teeth were included based on clinical and radiographic criteria. Inclusion criteria were: (1) non-vital immature permanent teeth, (2) presence of open apex with radiographic evidence of periapical radiolucency, and (3) absence of systemic illness. Teeth with root fractures, internal/external resorption, or severe periodontal involvement were excluded.

## Procedure:

All procedures were performed under rubber dam isolation. Access opening was carried out using sterile burs. Minimal instrumentation was performed to preserve the remaining dentinal walls. The canal was gently irrigated with 1.5% sodium hypochlorite (NaOCl), followed by 17% EDTA and saline. The canal was dried with sterile paper points. A triple antibiotic paste (ciprofloxacin, metronidazole, and minocycline) was placed as an intra-canal medicament for two weeks. After removal of the medicament, bleeding was induced by over-instrumentation beyond the apex using a sterile K-file. Instead of allowing the canal space to fill with blood directly, blood was collected from the fresh extraction socket or adjacent surgical site and transferred into the canal to act as the scaffold. The canal orifice was sealed with a 3 mm MTA plug, and the access cavity was restored with resin-modified glass ionomer cement followed by composite resin.

## Follow-up and assessment:

Patients were followed at 3, 6, and 12 months postoperatively. Clinical outcomes such as absence of pain, swelling, sinus tract, and response to percussion were recorded. Radiographic outcomes including resolution of periapical radiolucency, root lengthening, and apical closure were evaluated by two independent blinded endodontists.

## Results:

Out of the 20 patients enrolled in the research, 18 completed the full 12-month follow-up. Two were lost to follow-up after the 3-month review and were excluded from final analysis. All 18 teeth showed clinical resolution of symptoms by the 3rd month, with no reports of pain, swelling, or sinus tract formation throughout the observation period. Radiographic evaluation revealed progressive healing of periapical lesions, apical closure, and root development over the follow-up visits. By the 12-month review, 15 teeth (83.3%) showed complete resolution of the periapical radiolucency, and 13 teeth (72.2%) demonstrated continued root development with apical closure. Table 1 (see PDF) shows the distribution of clinical findings at various intervals. Pain and swelling subsided in all patients by the 3rd month. None of the teeth tested positive to percussion or showed sinus tract formation at any follow-up. Table 2 (see PDF) outlines the radiographic parameters. Progressive periapical healing was observed, with complete healing in 83.3% of cases by 12 months. Continued root development was seen in 72.2% of the treated teeth, while 5.5% showed no appreciable radiographic changes. These findings indicate that the use of alveolar-derived autologous blood clot as a scaffold in regenerative endodontics can result in favorable clinical and radiographic outcomes, including high rates of periapical healing and continued root development within a 12-month period.

## Discussion:

The present observational research evaluated the outcomes of using alveolar-derived autologous blood clot as a scaffold for RET in immature necrotic permanent teeth. The findings demonstrated that this simple, biologically derived scaffold can facilitate both clinical resolution of symptoms and radiographic signs of healing, including periapical lesion reduction, apical closure and root lengthening within 12 months of follow-up. The rationale behind using a blood clot as a scaffold lies in its natural composition. Blood clots are rich in fibrin, platelets and growth factors such as PDGF, TGF-β and VEGF, all of which contribute to angiogenesis, stem cell recruitment and tissue regeneration [11]. By utilizing blood collected from an alveolar socket, the present approach offered a more controlled and concentrated source of scaffold material compared to random bleeding induced within the canal, which may be inconsistent or inadequate in some cases [12]. This technique further reduces the need for expensive additives like PRP or PRF and simplifies the procedural workflow. The observed outcomes in this research such as 83.3% resolution of periapical lesions and 72.2% evidence of apical closure are in line with previous reports documenting the biological potential of scaffold-based regenerative procedures. These results support the hypothesis that creating a stable and biologically active scaffold within the canal environment can allow stem cells from the apical papilla to differentiate and contribute to tissue regeneration [13]. Root lengthening in 72.2% of cases further suggests that the regenerative process is not merely a reparative event but may mimic aspects of natural root development. However, the variability in outcomes, such as lack of response in one case (5.5%), could be attributed to factors such as individual healing potential, inadequate scaffold stability, or bacterial remnants within the canal system. Additionally, the absence of histologic confirmation remains a limitation in most clinical studies, including the present one. Regeneration is inferred from radiographic and clinical outcomes but not confirmed through tissue analysis [14]. These results align with evidence showing that blood clot scaffolds achieve clinical and radiographic outcomes comparable to PRP and PRF in apical closure, root development, and periapical healing. Although platelet concentrates may slightly improve vitality response or root thickness, overall success rates are similar. This supports the alveolar-derived autologous blood clot as a practical, biologically sound, and cost-effective scaffold for regenerative endodontics in immature necrotic permanent teeth [15, 16, 17, 18, 19- 20].

## Conclusion:

The potential of alveolar-derived autologous blood clot as an effective scaffold in RET for immature necrotic permanent teeth is shown. Being autologous, easily available, and biologically compatible, it offers a cost-effective and practical alternative to synthetic or platelet-derived scaffolds. The results affirm its clinical utility as a scaffold in facilitating biological root maturation in regenerative endodontics.
